# Correction: Wu, H.; et al. A Purified Aspartic Protease from *Akkermansia Muciniphila* Plays an Important Role in Degrading Muc2. *Int. J. Mol. Sci.* 2020, *21*, 72

**DOI:** 10.3390/ijms22063122

**Published:** 2021-03-18

**Authors:** Xin Meng, Wencheng Wang, Tianqi Lan, Wanxin Yang, Dahai Yu, Xuexun Fang, Hao Wu

**Affiliations:** 1 Key Laboratory for Molecular Enzymology and Engineering of Ministry of Education, College of Life Science, Jilin University, 2699 Qianjin Street, Changchun 130012, China; mengxin17@mails.jlu.edu.cn (X.M.); w1148893960@163.com (W.W.); SkyQiii@163.com (T.L.); yinglang6918@126.com (W.Y.); yudahai@jlu.edu.cn (D.Y.); 2 Vascular Biology Program, Department of Surgery, Boston Children’s Hospital and Harvard Medical School, Boston, MA 02115, USA

The authors wish to make the following corrections to this paper [1]:

We have recently found that during the image processing and assembly of Figure 7C, an incorrect immunofluorescence image was inadvertently selected for the Amuc_1434* degradation of Muc2. This was due to the fact that a different magnification was used before adding the scale bars, which caused the error to be overlooked between the control group and experimental group images. Therefore, a different graphical abstract is now also provided. 

The correct panel of Figure 7C is given below (Figure 1). This correction of Figure 7C does not affect any results and conclusions presented in the paper.

The authors would like to apologize for any inconvenience caused to the readers by these changes.

## Figures and Tables

**Figure 1:**
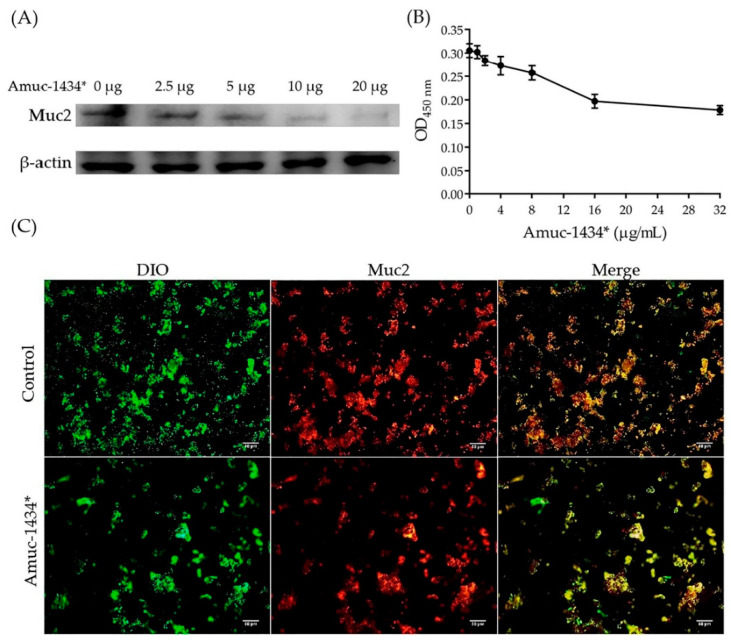
Amuc_1434* degradation of Muc2. (**A**) Western blot analysis of degradation of Muc2 by Amuc_1434* at different Amuc_1434* concentrations (loading protein were 0, 2.5, 5, 10 and 20 µg, respectively). (**B**) Enzyme-linked immunosorbent assay (ELISA) analysis of remaining Muc2 after Amuc_1434* degradation at 450 nm. Triplicate samples were measured and error bars represented standard deviation of the data. (**C**) Immunofluorescence analysis of the Muc2 degradation ability of Amuc_1434*. The membrane was shown in green with 3,30-dioctadecyloxacarbocyanine perchlorate (DIO). Muc2 was labeled in red. Scale bar = 50 µm.
